# Supplementary material for: Spectral signatures of the surface anomalous Hall effect in magnetic axion insulators
Source: Nat Commun. 2021 Jun 10;12:3524. doi: 10.1038/s41467-021-23844-z (PMC8192549; doi:10.1038/s41467-021-23844-z)
Supplement: Supplementary file 1 — Supplementary Information [file 41467_2021_23844_MOESM1_ESM.pdf]

Supplementary Information

Spectral signatures of the surface anomalous Hall effect in magnetic axion insulators

Mingqiang Gu[1], Jiayu Li[1], Hongyi Sun[1], Yufei Zhao[1], Chang Liu[1], Jianpeng Liu[2,3*], Haizhou Lu[1] and Qihang Liu[1,4,5,*]

[1]*Shenzhen Institute for Quantum Science and Engineering (SIQSE) and Department of Physics, Southern University of Science and Technology, Shenzhen 518055, China*

[2]*School of Physical Science and Technology, ShanghaiTech University, Shanghai, 200031, China*

[3]*ShanghaiTech laboratory for topological physics, ShanghaiTech University, Shanghai, 200031, China*

[4]*Guangdong Provincial Key Laboratory for Computational Science and Material Design, Southern University of Science and Technology, Shenzhen 518055, China*

[5]*Shenzhen Key Laboratory of for Advanced Quantum Functional Materials and Devices, Southern University of Science and Technology, Shenzhen 518055, China*

M. G. and J. L. contributed equally to this work.

[*]Emails: liujp@shanghaitech.edu.cn; liuqh@sustech.edu.cn

Supplementary Note 1. k·p model of the multilayer topological heterostructure

In the absence of inter-bilayer hopping and exchange, the multilayer model Eq. (1) in the main text reduces to a k_z-independent form

$$H_{\text{intra}}(k_\parallel) = \begin{pmatrix} \hbar v_f(\boldsymbol{\sigma}\times\mathbf{k})_z & m_k & 0 & 0 \\ m_k & -\hbar v_f(\boldsymbol{\sigma}\times\mathbf{k})_z & t_{IB}^0 & 0 \\ 0 & t_{IB}^0 & \hbar v_f(\boldsymbol{\sigma}\times\mathbf{k})_z & m_k \\ 0 & 0 & m_k & -\hbar v_f(\boldsymbol{\sigma}\times\mathbf{k})_z \end{pmatrix}, \tag{1}$$

where $m_k = \Delta - Bk_\parallel^2$. Since the inter-bilayer hopping terms couple the first and last block in the present space, only the effective interactions between these two blocks are essential while considering the inter-bilayer hopping. We take the eigenvector of H_{intra} as $\psi = (\psi_1, \psi_2, \psi_3, \psi_4)^T$. The Schrodinger's equation of H_{intra} reads

$$\begin{array}{rcl} \hbar v_f(\boldsymbol{\sigma}\times\mathbf{k})_z\psi_1 + m_k\psi_2 & = & E\psi_1, \\ m_k\psi_1 - \hbar v_f(\boldsymbol{\sigma}\times\mathbf{k})_z\psi_2 + t_{IB}^0\psi_3 & = & E\psi_2, \\ t_{IB}^0\psi_2 + \hbar v_f(\boldsymbol{\sigma}\times\mathbf{k})_z\psi_3 + m_k\psi_4 & = & E\psi_3, \\ m_k\psi_3 - \hbar v_f(\boldsymbol{\sigma}\times\mathbf{k})_z\psi_4 & = & E\psi_4. \end{array} \tag{2}$$

Substituting ψ_2 and ψ_3 into the first and last equations, we derive a self-consistent matrix equation:

$$\begin{pmatrix} \hbar v_f(\boldsymbol{\sigma}\times\mathbf{k})_z & (t_{IB}^0)^{-1}\left(E^2 - m_k^2 - \hbar^2 v_f^2 k_\parallel^2\right) \\ (t_{IB}^0)^{-1}\left(E^2 - m_k^2 - \hbar^2 v_f^2 k_\parallel^2\right) & -\hbar v_f(\boldsymbol{\sigma}\times\mathbf{k})_z \end{pmatrix}\begin{pmatrix}\psi_1\\ \psi_4\end{pmatrix} = E\begin{pmatrix}\psi_1\\ \psi_4\end{pmatrix}. \tag{3}$$

The eigen energies are solved as

$$\varepsilon_{\pm,\alpha} = \pm\sqrt{m_k^2 - \hbar^2 v_f^2 k_\parallel^2 + \frac{1}{2}|t_{IB}^0|[|t_{IB}^0| + (-1)^\alpha \Lambda_k]}, \tag{4}$$

where $\Lambda_k = \sqrt{(t_{IB}^0)^2 + 4m_k^2 - 8\hbar^2 v_f^2 k_\parallel^2}$ and $\alpha = 1,2$. We choose the energy pair with $\alpha = 1$ to capture the property of phase transition since the other pair with $\alpha = 2$ never close the gap. Therefore, the effective Hamiltonian in the basis of $(\psi_1, \psi_4)^T$ is reached

$$H_{\text{eff}}(k_\parallel) = s_z \hbar v_f(\boldsymbol{\sigma}\times\mathbf{k})_z + m_{k,\text{eff}} s_x, \tag{5}$$

with the effective mass term $m_{k,\text{eff}} = \text{sgn}(t_{IB}^0)(|t_{IB}^0| - \Lambda_k)/2$. Expanding $m_{k,\text{eff}}$ up to the square of $k_\parallel$ and adding magnetic exchange, we find that those two bands of the bilayer take the form of Bernevig-Hughes-Zhang model

$$H_{\text{eff}}(k_\parallel) = \hbar v_f s_z(\boldsymbol{\sigma}\times\mathbf{k})_z + \left[\Delta_{\text{eff}}(t_{IB}^0) - B_{\text{eff}}(t_{IB}^0)k_\parallel^2\right]s_x + M\sigma_z. \tag{6}$$

Here $\Delta_{\text{eff}}(t_{IB}^0) = \text{sgn}(t_{IB}^0)(|t_{IB}^0| - \Lambda_{k=0})/2$ and $B_{\text{eff}}(t_{IB}^0) = -2\text{sgn}(t_{IB}^0)\left(\hbar^2 v_f^2 + \Delta B\right)/$

$\Lambda_{k=0}$, in which $\Delta_{\text{eff}}/B_{\text{eff}} > 0$ demarcates the no trivial phase in this quasi-2D bilayer even if $\Delta/B < 0$.

Afterwards we turn on the inter-bilayer hopping t_{IB} with periodic boundary on z direction. One directly finds the dispersions

$$\varepsilon_{\pm\eta}(k) = \pm\sqrt{\hbar^2 v_f^2 k_\parallel^2 + M^2 + m'^2_{k,\text{eff}} + t_{IB}^2 + 2m'_{k,\text{eff}} t_{IB} \cos(k_z D) + (-1)^\eta 2|M|\delta_k}, \quad (7)$$

with $\eta = 0,1$, $m'_{k,\text{eff}} = \Delta_{\text{eff}}(t_{IB}^0) - B_{\text{eff}}(t_{IB}^0)k_\parallel^2$, and $\delta_k = \sqrt{m'^2_{k,\text{eff}} + t_{IB}^2 + 2m'_{k,\text{eff}} t_{IB} \cos(k_z D)}$. The dispersions share the form with Eq. (3) in Ref. 1 but with a momentum-dependent intralayer hopping term instead, distinguishing the 2D normal insulating phase and QSH phase. Bands near $E_f = 0$, i.e. $\varepsilon_{s1}(k)$, touch each other at $k = (0,0,k_0)$ as

$$k_0 D = \arccos\frac{M^2 - \Delta_{\text{eff}}^2(t_{IB}^0) - t_{IB}^2}{2\Delta_{\text{eff}}(t_{IB}^0)t_{IB}}, \quad (8)$$

only with the inequation $(|\Delta_{\text{eff}}(t_{IB}^0)| - |t_{IB}|)^2 < M^2 < (|\Delta_{\text{eff}}(t_{IB}^0)| + |t_{IB}|)^2$ satisfied. Critical boundaries of distinct phases in Fig. 1 of the main text are settled by the inequation with parameters $\hbar v_f = 2.6\ \text{eV}\cdot\text{Å}, \Delta = 0.019\ \text{eV}, B = -40\ \text{eV}\cdot\text{Å}^2, d_0 = 2.7\ \text{Å}, t_{IB}^0 = 0.033\ \text{eV}$, and $\alpha = 4.6$ from model Hamiltonians of bulk and thin film of Bi_2Te_3[2, 3].

Supplementary Note 2. The fragile topology of the 3D T-broken QSH phase

The parity distribution at inversion-invariant k-points of the 3D T-broken QSH insulator can be viewed as nearly isolated layer stacking of 2D T-broken QSH insulators. Similar to the other phases (axion insulator, Weyl semimetal, and 3D Chern insulator), the 3D T-broken QSH phase is unable to decompose into the symmetric Wannier functions. However, such a Wannier obstruction for the QSH phase can be removed by adding a set of trivial elementary band representations (EBRs).

To check the fragile nature of the 3D T-broken QSH phase, we construct the EBRs, i.e., the Wannier representations via putting p-orbital at different sites: A(0,0,0), B(1/2,0,0), C(0,1/2,0), D(1/2,1/2,0), E(0,0,1/2), F(1/2,0,1/2), G(0,1/2,1/2), and H(1/2,1/2,1/2), as shown in Supplementary Fig. 1(a). For each EBR, the parity distribution at inversion-invariant k-points is directly found. For instance, the parity $\pi_{\mathbf{k}}$ of the EBR $p@B$ is $\pi_\Gamma = \pi_Y = \pi_Z = \pi_V = +1$, and $\pi_X = \pi_M = \pi_U = \pi_W = -1$ [See Supplementary Fig. 1(b) for the definition of the high-symmetric k-points], where the p-orbital is invariant under T_xP operation with T_x the translation operators along x direction. The parity distribution of eight EBRs is listed in Supplementary Table 1.

As shown in Fig. 1 in the main text, the parity distribution of the 3D T-broken QSH phase is $(\pi_\Gamma, \pi_X, \pi_Y, \pi_M, \pi_U, \pi_V, \pi_W) = (-2, +2, +2, +2, -2, +2, +2, +2)$. With the EBRs as the building blocks with parities as listed in Supplementary Table 1, one can decompose the 3D T-broken QSH state as $p@B + p@C + p@D - p@A$. In other words, such a representation does not admit any Wannier representation unless an additional trivial representation $p@A$ is considered together, as sketched in Supplementary Fig. 1(d).

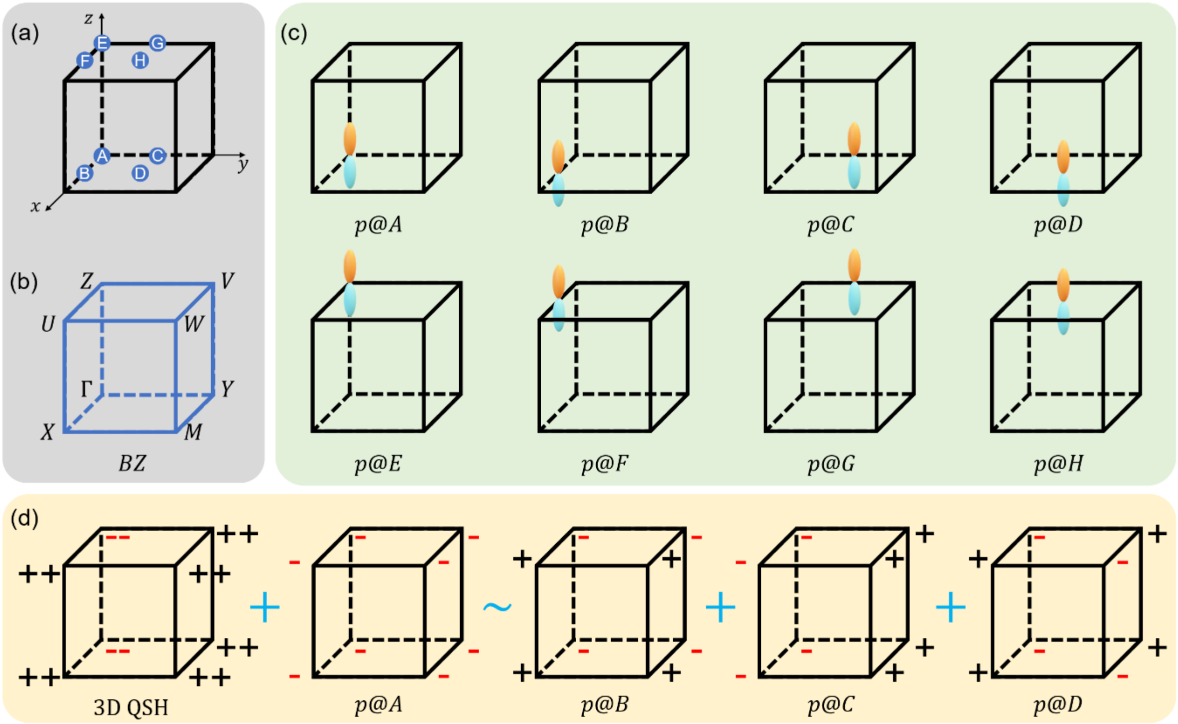

Supplementary Figure 1. Construction of the elementary band representations. (a) Eight sites in a three-dimensional cell with inversion symmetry. (b) The Brillouin zone with eight inversion-invariant k-points. (c) Eight elementary band representations are constructed with a p-orbital state at one of the eight sites of (a). (d) The decomposition of the 3D T-broken QSH phase adding a trivial $p@A$ representation.

Supplementary Table 1. The parity distribution of eight EBRs at inversion-invariant k-points.

$\pi_{\mathbf{k}}$	Γ	X	Y	M	Z	U	V	W
$p@A$	−	−	−	−	−	−	−	−
$p@B$	−	+	−	+	−	+	−	+
$p@C$	−	−	+	+	−	−	+	+
$p@D$	−	+	+	−	−	+	+	−
$p@E$	−	−	−	−	+	+	+	+
$p@F$	−	+	−	+	+	−	+	−
$p@G$	−	−	+	+	+	+	−	−
$p@H$	−	+	+	−	+	−	−	+

Supplementary Note 3. Electronic structures of different topological phases in $MnBi_2Te_4$

The electronic band structures for different topological nontrivial phases depicted in the phase diagram in the main text are shown in Supplementary Fig. 2. The pristine $MnBi_2Te_4$ is an axion insulator with a band gap of ~100 meV, while when the inter-bilayer hopping integral is quenched it turns into a 3D Chern insulator phase, which has exactly the same band structure of a bilayer $MnBi_2Te_4$. A Weyl semimetal phase can be obtained by applying 1% tensile strain on the lattices of $MnBi_2Te_4$, the band structure of which is shown in Supplementary Fig. 2(c). Two Weyl nodes are located at $k_z = \pm 0.018 \cdot \frac{2\pi}{c}$. The corresponding band structures for the slabs built with these phases are shown in Supplementary Fig. 2(d-f).

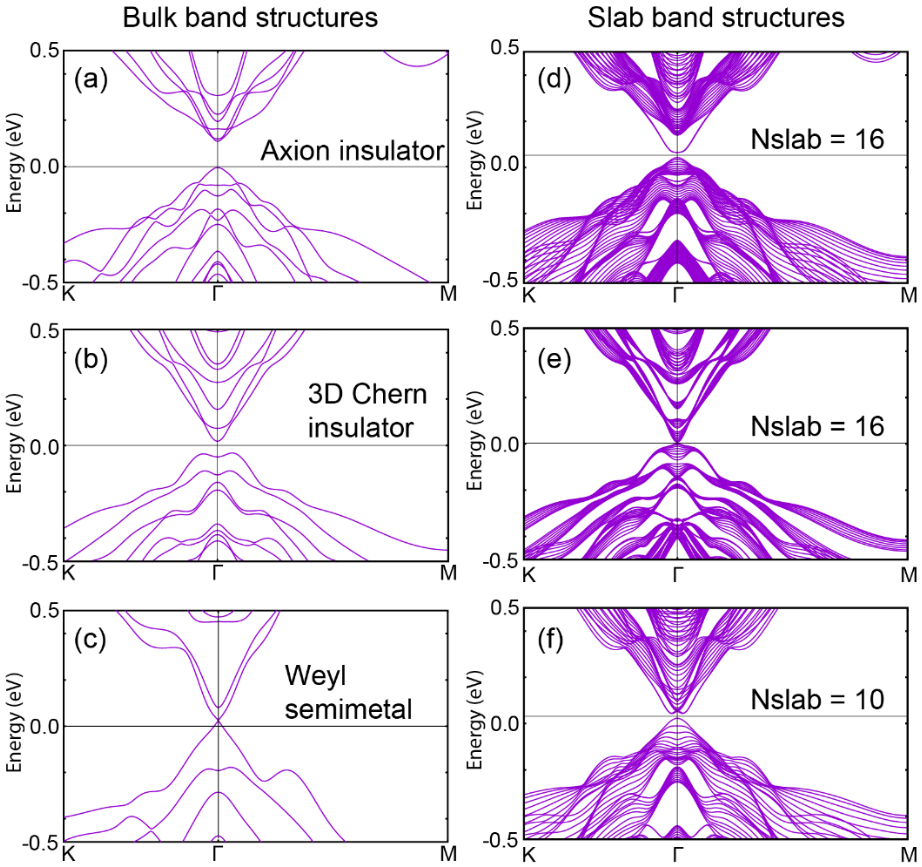

Supplementary Figure 2. The bulk band structures for FM $MnBi_2Te_4$ in (a) axion insulator, (b) 3D Chern insulator and (c) Weyl semimetal phases. The band structures for the corresponding slabs are plotted in (d-f), the layered Chern numbers of which are shown in Fig. 2 in the main text.

Supplementary Note 4. Locality of the half-quantized surface AHC

To further demonstrate that the half-quantized AHC of an axion insulator is a local property at the gapped surface, we consider a 16-layer slab of FM $MnBi_4Te_7$, which is insulating for $MnBi_2Te_4$ termination but metallic for Bi_2Te_3 termination [see Supplementary Fig. 3(a)]. We find that as long as E_f locates within the surface gap of the $MnBi_2Te_4$ termination, the corresponding surface AHC would stay around 1/2 at the gapped surface. On the other hand, the surface AHC with the metallic Bi_2Te_3 termination varies with different choice of E_f. As shown in Supplementary Fig. 3(b), if we move E_f up by 0.01 eV, $\mathbb{C}(l)$ at the Bi_2Te_3-terminated surface is dropped by 0.05, while the $MnBi_2Te_4$-terminated surface still exhibits the half quantization. Further elevating E_f cuts the conduction band of MnBi2Te4 termination and thus violates the half quantization by decreasing the surface AHC, denoted by the arrow in Supplementary Fig. 3(b).

In addition, we also build symmetric slabs with $(4n + 1)$ VdW layers which both surfaces terminated by the Bi_2Te_3 to check whether half quantization can be realized in the Bi_2Te_3-termination. Since there is no net magnetization of these slabs, the total Chern number should be zero. DFT calculation proves that such a slab has a global gap, thus the layered Chern numbers for the two surfaces are well defined and should be symmetrically canceled out. Supplementary Fig. 3(c,d) shows the geometry of these slabs, electronic band structure and the resulting layered Chern number.

The above results show the locality of the half-quantized surface AHC.

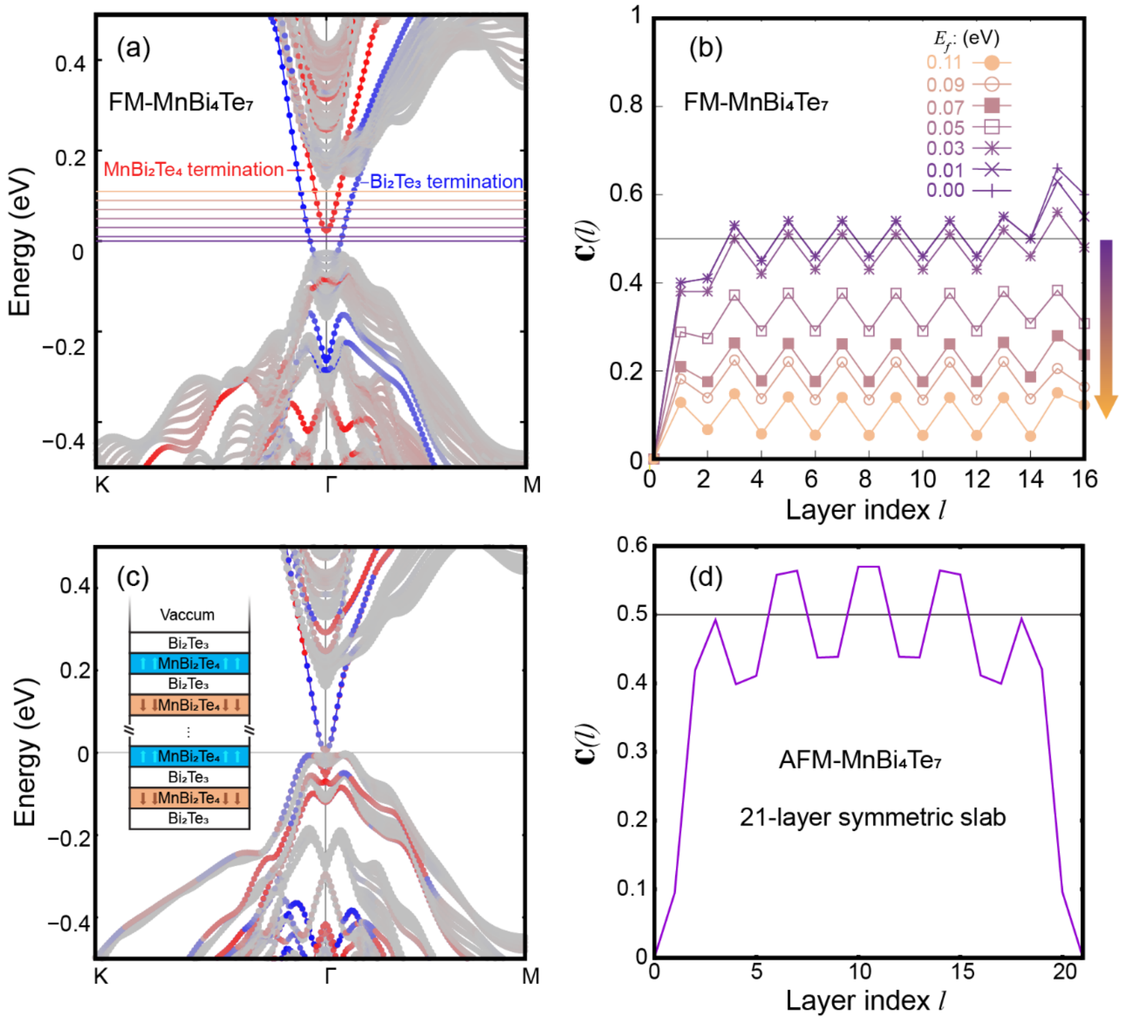

Supplementary Figure 3. (a) The electronic band structure for FM-$MnBi_4Te_7$. The horizontal lines denote different positions of the Fermi energy. (b) The integrated Chern number computed for FM-$MnBi_4Te_7$. Different curves correspond to different E_f denoted in (a). (c) The electronic band structure for a symmetric slab made of AFM-$MnBi_4Te_7$. Both surfaces are Bi_2Te_3-terminated, shown in the inset. (d) The integrated Chern number computed for the symmetric AFM-$MnBi_4Te_7$ slab.

Supplementary Note 5. Calculations of the surface state and chiral hinge state

The surface state is computed by iterative Green's function implemented in WannierTools package[4]. The spectral function, i.e. imaginary part of the Green's function is then plotted in Fig. 3 and 4 in the main text.

For the hinge states, two methods have been developed and the results are cross-checked. The first method directly computes the spectral function for the hinge along a particular direction (in our case, along y). x and z directions are semi-infinite. The bi-semi-infinite geometry of the system is plotted in Supplementary Fig. 5. In order to calculate the hinge states, the tight-binding Hamiltonian of a unit cell (h_0) is computed with the WANNIER90 code interfaced to VASP. Then the Hamiltonian for a supercell (H_0) with the thickness of a certain principle layers (PL's) along x and z directions is constructed. The electron hopping integrals along x (H_1^x) and z (H_1^z) directions are retained only between the adjacent supercells. One can then get Eq. (4-6) in Methods session. Let Σ_R be the self-energy of the interactions between the 'hinge' and the other layers in the x and z directions. We have,

$$G_R(k_y, \omega) = [(\omega + i\eta) - H_0 - \Sigma_R]^{-1} \tag{9}$$

An approximation is then made that

$$\Sigma_R = H_1 g_R H_1^\dagger \approx H_1^x g_x H_1^{x\dagger} + H_1^z g_z H_1^{z\dagger} \tag{10}$$

where g_x and g_z are the surface Green's function along the two directions, which can be solved with the standard iteration scheme.

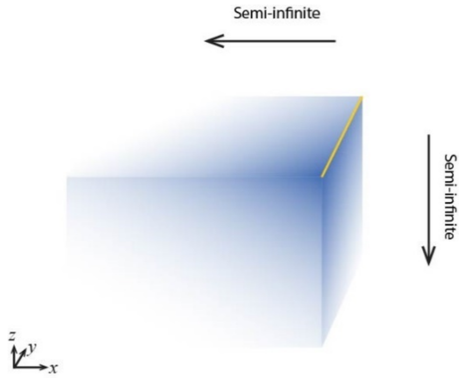

Supplementary Figure 4. The bi-semi-infinite geometry of the hinge states calculation.

The first method described above gives the spectral function right at the hinge. In order to investigate the spectral function away from the hinge, a slab that is finite in the x-direction, semi-infinite in the z-direction and infinite in the y-direction is used, plotted in Supplementary Fig. 5(a). In this sense, k_y is a well-defined quantum number. The thickness convergence along x has been tested and we find that 20-unit-cell is thick enough to decouple the hinge state and the top surface state. The spectral functions at the two hinges and the center of the top surface, denoted as B, C and D in Supplementary Fig. 5(a), are computed. These are the same spots computed for as in the Fig. 5(b-d) in the main text, but in a wider energy scale window. It is clear that the hinge states at B and D carry reverse momenta, and therefore are chiral. While at the position of C the hinge states vanish, and it reveals the gapped top surface. The spots for the hinge states plotted in Fig. 5(g-i) in the main text are also denoted in Supplementary Fig. 5.

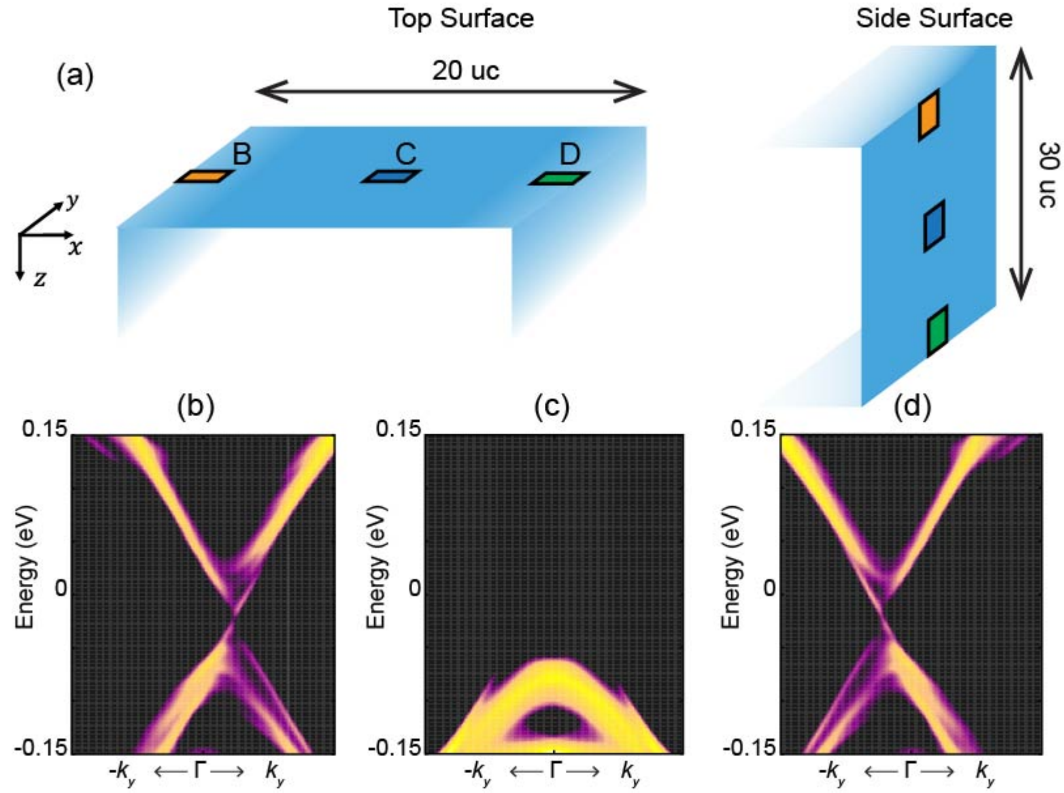

Supplementary Figure 5. Details for the hinge state calculation. (a) A schematic picture showing the model we used for hinge state calculation. The spectral functions for the three colored spots at the top surface denoted by B/C/D are plotted in (b), (c) and (d) respectively, the same spots computed for as in the Fig. 4(b-d) in the main text. The right panel in (a) denotes the spots calculated for the side surface, as plotted in Fig. 4(g-i) in the main text.

Supplementary Note 6. Origin of the hinge state

The in-band hinge states essentially arise from the difference of the surface anomalous Hall conductivities of the two surfaces linked by the hinge. Let us consider the situation of two T-broken surfaces connected by a hinge and the Fermi level is fixed at E_F cutting through the surface states for at least one surface. Then one can calculate the surface anomalous Hall conductivities for both surfaces, which are denoted by σ_1 and σ_2 [see schematic illustration in Supplementary Fig. 6(a)]. If an electric field E_y is applied along the y direction, the anomalous Hall current densities are generated at both surfaces with $j_1 = \sigma_1 E_y$ and $j_2 = \sigma_2 E_y$, changing from j_1 to j_2 as the current passes through the hinge. Since the current density must be conserved, the hinge has to carry the extra current density $j_1 - j_2$. Thus, there has to be chiral modes localized at the hinge carrying the extra anomalous Hall current density from the surface, which means that the spectral weights of the left-moving modes and right-moving modes at the hinge have to be unequal. This gives rise to the chiral in-band hinge modes. A special situation is that one of the two surfaces is gapless and the other surface is gapped, then no matter where E_F is, the chiral hinge mode as a signature of the difference of the surface anomalous Hall effects, has to be embedded into the gapless surface state, which is exactly the case of the hinge mode in AFM $MnBi_2Te_4$ [Figs. 3(b) and 3(c)].

When E_F is in the gap of the surface states at both surfaces, the surface anomalous Hall conductivity is proportional to the bulk orbital magnetoelectric coupling, which is half quantized in axion insulators. Then one has to dinstiguish the case that the two surfaces have opposite surface AHCs $\pm 1/2$ or the same AHCs, in the former there is a chiral in-gap hinge mode [② in Fig. 3(f)], and in the latter there is no hinge mode [④ in Fig. 3(f)].

Previous experiments on thin films of magnetic TI $Cr_x(Bi_{1-y}Sb_y)_{2-x}$ resulted in dual edge modes with a step change in Chern number $\Delta C = 2$ at the domain wall, manifesting the Chern numbers $C = \pm 1$ for each domain[5]. In stoichiometric $MnBi_2Te_4/(Bi_2Te_3)_n$, on the other hand, an effective surface Hamiltonian with a domain wall extending along the x axis: $H(x,y) = -iv(\partial_y \sigma_x - \partial_x \sigma_y) - sgn(y) M_z \sigma_z$. Such a Hamiltonian hosts a single chiral eigenmode with linear dispersion $E = vk_x$ localized at the domain wall, indicating a discontinuous change

$\Delta C = 1$. Since the two domains are related by time-reversal operation, this single chiral mode indicates the opposite surface Chern number $\pm 1/2$ for each domain.

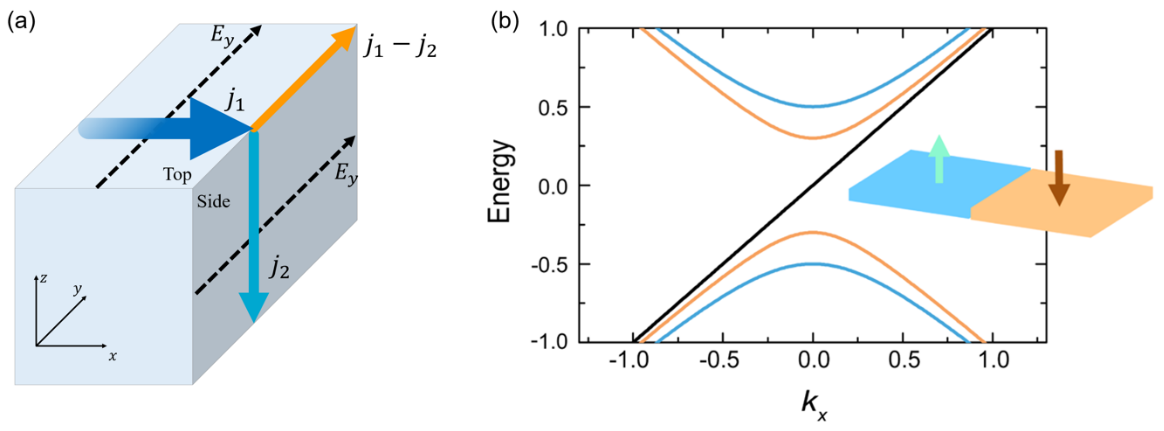

Supplementary Figure 6. (a) Schematic plot of the in-band hinge modes arise from the difference of the surface anomalous Hall conductivities of the two connecting surfaces. (b) Energy spectrum with a single chiral mode at the surface of $MnBi_2Te_4/(Bi_2Te_3)_n$ with a magnetic domain wall.

Supplementary Note 7. Comparison between magnetic axion insulator and trivial insulator

For a magnetic system, the electronic band will respond to the magnetic field in the way that the weights on different sides are different, leading to a chiral state. Once the Fermi level cuts across this band, i.e. in the metal case, it immediately leads to a Hall current, a direct result caused by the asymmetric left- and right-moving modes. In this sense, such a chiral in-band hinge state is a generic effect for any magnetic system. Moreover, in an axion insulator, this imbalanced chiral in-band state is much more significant than that in a trivial insulator owing to the intrinsic additional 1/2 AHC. To verify this, we have compared the ferromagnetic axion insulator $MnBi_2Te_4$ and trivial insulator $MnSb_2Te_4$ and found the asymmetric effects in the in-band chiral hinge are much weaker in $MnSb_2Te_4$ than that in $MnBi_2Te_4$, see the figure below. Therefore, such a large chiral in-band hinge state is the signature of an axion insulator.

By comparing the in-band states in an axion insulator and the trivial insulator, one immediately finds two major differences: a) The asymmetry of the valence band is more significant in the axion insulator; b) The shift of the center of the Dirac cone in the trivial insulator is negligible, while that in the axion insulator is prominent, towards the $+k_y$ direction. In fact, the position of the Dirac cone in the hinge states is exactly the same position for the side surface Dirac cone in the axion insulator, i.e. Figure 4(f) in the main text.

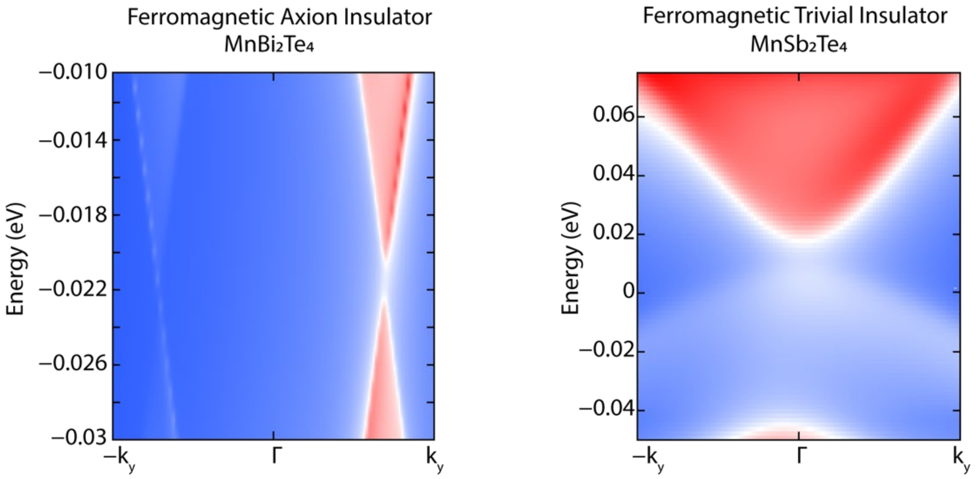

Supplementary Figure 7. Hinge states for ferromagnetic axion insulator $MnBi_2Te_4$ and trivial insulator $MnSb_2Te_4$. The chiral nature of the in-band hinge state is much more significant in the axion insulator $MnBi_2Te_4$.

Supplementary Note 8. Two types of the surface gap in $MnBi_2Te_4/(Bi_2Te_3)_n$

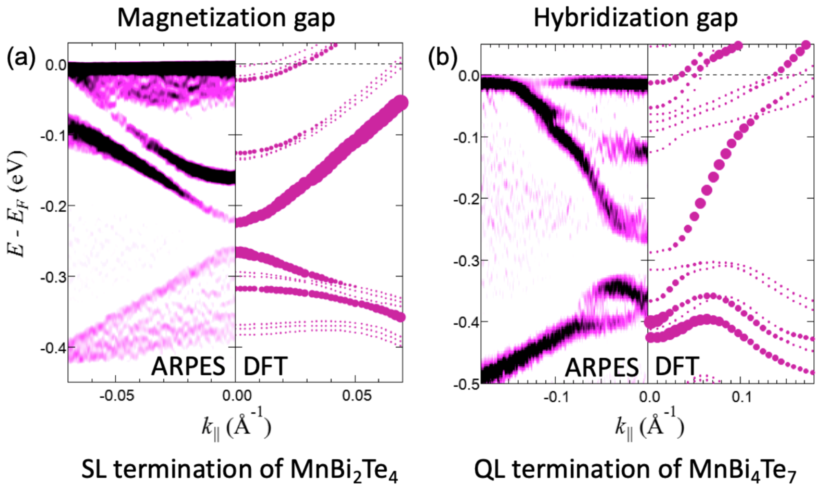

Supplementary Figure 8. Comparison of band structures between ARPES and DFT results for the (001) surface of (a) $MnBi_2Te_4$ with slightly Sb doping and (b) the Bi_2Te_3-termination of $MnBi_4Te_7$. The ARPES results are taken from Ref. 6 and 7, respectively.

To describe two types of surface gap in the system [Fig. 3(a) and (b) in the main text], we construct a uniform surface ***k·p*** model Hamiltonian to extract the central information of the magnetization- and hybridization-induced gap[7]. Taking Dirac bands, Rashba bands, hexagonal warping, and hybridization between Dirac and Rashba bands into account, we build the Hamiltonian in the following:

$$H_{\mathrm{sur}}(k_{\parallel}) = \begin{pmatrix} H_k^1 & T \\ T & H_k^2 \end{pmatrix}. \quad (11)$$

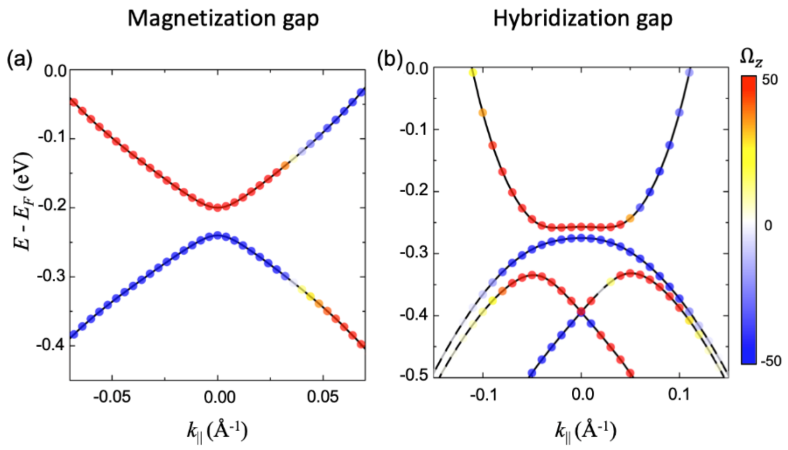

Supplementary Figure 9. Band structures of the (0001) surface of (a) $MnBi_2Te_4$ and (b) the Bi_2Te_3 termination of $MnBi_4Te_7$ simulated by surface $k{\cdot}p$ model with color map on Berry curvature.

In Eq. (11), $H_k^1 = \varepsilon_k^D + v_F^D(\boldsymbol{\sigma} \times \mathbf{k})_z + \left[m_z^D + \lambda k_x\left(k_x^2 - 3k_y^2\right)\right]\sigma_z$ denotes the Dirac cone of the topmost surface $MnBi_2Te_4/(Bi_2Te_3)_n$ with a mass $m_z^D\sigma_z$ introduced to capture the surface gap observed in various conditions, e.g., Sb-doped $MnBi_2Te_4$[6]. $H_k^2 = \varepsilon_k^R + v_F^R(\boldsymbol{\sigma} \times \mathbf{k})_z + m_z^R\sigma_z$ describes a pair of Rashba bands with exchange field m_z^R capturing the dispersion from the second topmost layers. $\varepsilon_k^{D/R}$ and $v_F^{D/R}$ denote the parabolic dispersion and velocity of Dirac and Rashba bands, respectively, with λ and T the parameters of hexagonal warping and hybridization strength between the top two layers.

For n=0, i.e., $MnBi_2Te_4$, the surface dispersion can be mainly produced by the H_k^1 block thanks to the homogeneity of the top two layers. Supplementary Fig. 9(a) shows the surface bands simulating of the Sb-doped $MnBi_2Te_4$ with parameters: $\varepsilon_k^D = (-0.22 + k^2)$ eV, $\hbar v_F^D = 2.35$ eV·Å, $\lambda = 200$ eV·Å^3, and $m_z^D = 0.02$ eV. Otherwise, for n>0, the surface bands of the Bi_2Te_3 termination (topmost) above the $MnBi_2Te_4$ layer (second topmost) are represented by the full matrix Eq. (9). Surface bands of the Bi_2Te_3 termination of $MnBi_4Te_7$ are shown in Supplementary Fig. 9(b) with parameters: $\varepsilon_{\mathbf{k}}^D = (-0.38 + 3.6\,k^2)$ eV, $\hbar v_F^D = 2.05$ eV·Å, $\lambda = 200$ eV·Å^3, $m_z^D = 0$ eV, $\varepsilon_{\mathbf{k}}^R = (-0.28\ - 10\,k^2)$ eV, $\hbar v_F^R = 0$ eV·Å, $m_z^R = 0.01$ eV, and $T = 0.03$ eV.

In addition, the opposite Berry curvature $\Omega_z^i(k) = i\nabla_k \times \langle u_k|\nabla_k|u_k\rangle$ of bands above and below the gap shows the topological property of both the magnetization- and hybridization-induced gap, as displayed in Supplementary Fig. 9.

Supplementary References

1. Burkov AA, Balents L. Weyl Semimetal in a Topological Insulator Multilayer. *Physical Review Letters* **107**, 127205 (2011).
2. Liu C-X, Qi X-L, Zhang H, Dai X, Fang Z, Zhang S-C. Model Hamiltonian for topological insulators. *Phys Rev B* **82**, 045122 (2010).

3. Lu H-Z, Shan W-Y, Yao W, Niu Q, Shen S-Q. Massive Dirac fermions and spin physics in an ultrathin film of topological insulator. *Phys Rev B* **81**, 115407 (2010).
4. Wu Q, Zhang S, Song H-F, Troyer M, Soluyanov AA. WannierTools: An open-source software package for novel topological materials. *Computer Physics Communications* **224**, 405-416 (2018).
5. Yasuda K*, et al.* Quantized chiral edge conduction on domain walls of a magnetic topological insulator. *Science* **358**, 1311-1314 (2017).
6. Ma X-M*, et al.* Spectroscopic realization of large surface gap in a doped magnetic topological insulator. *arXiv*, 2004.09123 (2020).
7. Wu X*, et al.* Distinct Topological Surface States on the Two Terminations of MnBi4Te7. *Phys Rev X* **10**, 031013 (2020).
